# Mechanisms of impact and experiences of a person-centred transition programme for adolescents with CHD: the Stepstones project

**DOI:** 10.1186/s12913-021-06567-1

**Published:** 2021-06-10

**Authors:** Markus Saarijärvi, Lars Wallin, Philip Moons, Hanna Gyllensten, Ewa-Lena Bratt

**Affiliations:** 1 Institute of Health and Care Sciences, University of Gothenburg, Gothenburg, Sweden; 2 Department of Public Health and Primary Care, KU Leuven, Leuven, Belgium; 3 School of Education, Health and Social Studies, Dalarna University, Falun, Sweden; 4 Department of Paediatrics and Child Health, University of Cape Town, Cape Town, South Africa; 5 University of Gothenburg Centre for Person-Centred Care (GPCC), Sahlgrenska Academy, University of Gothenburg, Gothenburg, Sweden; 6 Department of Pediatric Cardiology, The Queen Silvia Children’s Hospital, Gothenburg, Sweden

**Keywords:** Adolescent, Chronic disease, Parent, Process evaluation, Qualitative research, Randomized controlled trial, Transition of care

## Abstract

**Background:**

During the past decade there has been some evaluation of transition programmes for adolescents with chronic conditions. However, this has rarely involved process evaluations focusing on mechanisms leading to outcomes, thus hampering implementation of these complex interventions. Our aim was to (I) describe adolescents’ and parents’ experiences of participating in a person-centred transition programme aiming to empower them in transition to adulthood and (II) explore the mechanisms of impact.

**Methods:**

A qualitative process evaluation was performed, embedded in a randomized controlled trial evaluating the effectiveness of a transition programme for adolescents with congenital heart disease in Sweden. A purposive sample of 14 adolescents and 12 parents randomized to the intervention group were interviewed after participation in the programme. Data were analysed deductively and inductively in NVivo v12.

**Results:**

Experiences of participation in the transition programme were generally positive. Meeting a transition coordinator trained in person-centred care and adolescent health and embarking on an educational process based on the adolescents’ prerequisites in combination with peer support were considered key change mechanisms. However, support to parents were not sufficient for some participants, resulting in ambivalence about changing roles and the unmet needs of parents who required additional support.

**Conclusions:**

Participants experienced increased empowerment in several dimensions of this construct, thus demonstrating that the transition programme was largely implemented as intended and the evidence-based behaviour-change techniques used proved effective in reaching the outcome. These findings can inform future implementation of transition programmes and illuminate challenges associated with delivering a complex intervention for adolescents with chronic conditions.

**Supplementary Information:**

The online version contains supplementary material available at 10.1186/s12913-021-06567-1.

## Background

Adolescence and young adulthood pose challenges for most individuals in the process of becoming an independent person. For young people living with chronic conditions (CC), there is an additional challenge in balancing the developmental tasks associated with adolescence, such as developing a sense of identity, planning for the future and gaining independence from parents. Moreover, the young person must also attain self-management skills to manage their health and care, develop healthy behaviours and cope with challenges related to their CC [1–4].

Transition programmes have been developed to support the transition to adulthood and transfer from paediatric to adult care. These programmes have been evaluated through randomized controlled trials (RCT), showing positive results in patient-reported outcomes (i.e. disease-related knowledge, self-efficacy and self-management) [5], as well as in reducing delay in transfer to adult care [6, 7]. However, none of these RCTs have published process evaluation studies explaining the mechanisms that led to these outcomes [8]. Indeed, transition programmes are complex interventions, consisting of multiple interacting components, targeting different organizational levels and requiring a change in behaviour on the part of those involved in delivering and interacting with the intervention. Process evaluations of these interventions are therefore essential. The objective of a process evaluation is to understand how and why an intervention works by assessing the implementation process, the contextual impact on this process and what change mechanisms were underlying in achieving (or not achieving) the outcome [9]. As process evaluation studies of transition programmes are lacking, these programmes remain ‘black boxes’ for which mechanisms are unknown, this hampering reproducibility and implementation of transitional care.

This paper reports on a process evaluation embedded in the STEPSTONES (Swedish Transition Effects Project Supporting Teenagers with chrONic mEdical conditionS) project, for which a person-centred transition programme for adolescents with congenital heart disease (CHD) was evaluated through an RCT [10, 11]. The primary outcome was patient empowerment, which is a resource relating to self-management and participation in care, aiming to make the adolescent an active partner in the decision-making process [12, 13]. A fundamental aspect of understanding the change created by a complex intervention arises through investigating the link between the programme activities and outcomes [9, 14]. By exploring change mechanisms we can achieve a deeper understanding of which components were more important and which components were redundant, thus facilitating reproducibility of the intervention into other settings. The aim of this paper therefore was to (I) describe adolescents and their parents’ experiences of participating in a person-centred transition programme aiming to empower them in transition to adulthood, and (II) to explore the potential mechanisms of impact from their perspective.

## Methods

### Design

A qualitative explorative design was employed. This study was part of a larger process evaluation study [11, 15]. The planning and reporting of this study followed the Consolidated Criteria for Reporting Qualitative studies (COREQ) checklist [16] (Additional file 1).

### The STEPSTONES transition programme

**Fig. 1:**
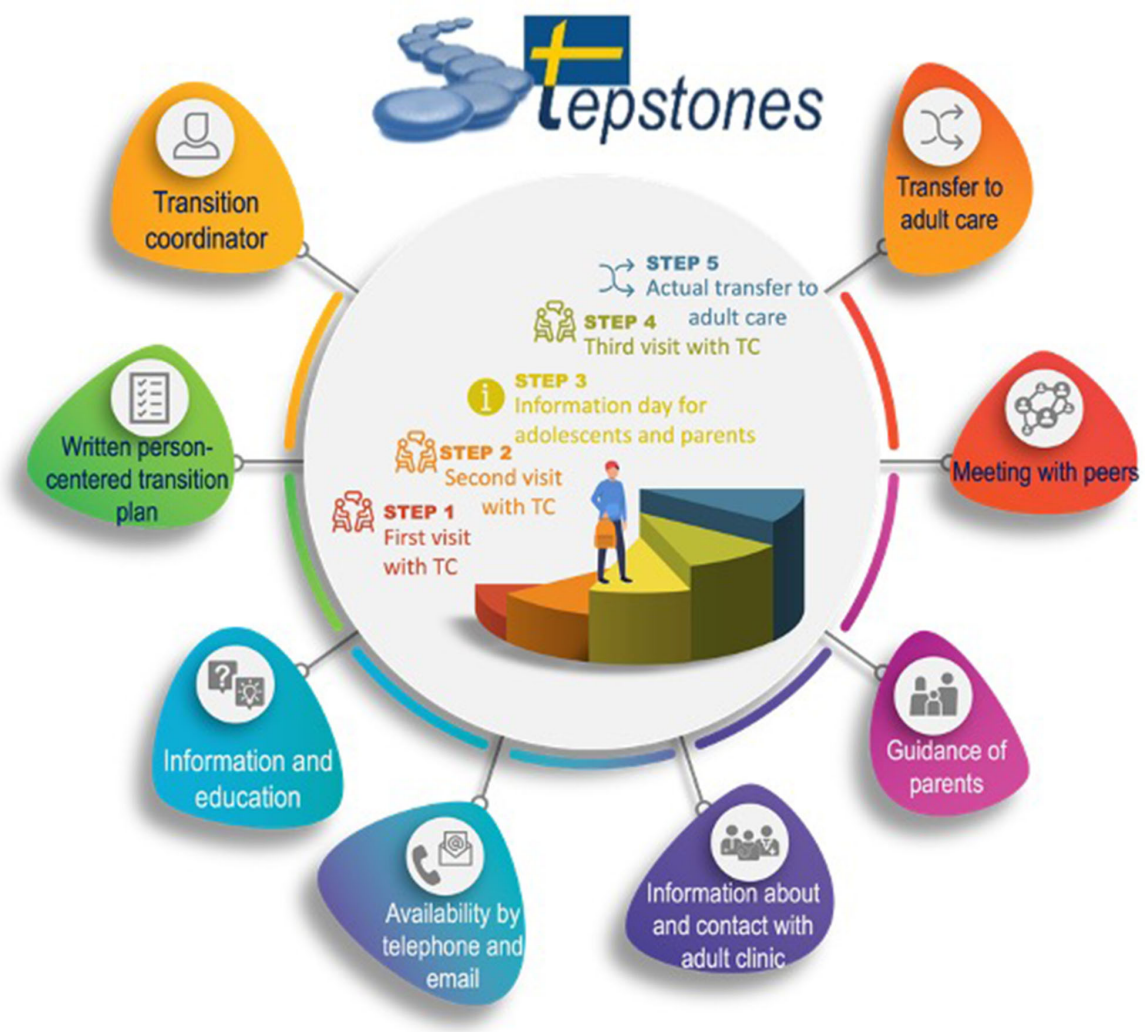
Components of STEPSTONES transition programme and implementation steps

Development of the transition programme followed the protocol of intervention mapping [17, 18]. The outcome of patient empowerment was chosen as it comprises five dimensions that are crucial to the adolescent’s ability to manage the transfer to adult care and transition to adulthood: 1) knowledge and understanding; 2) personal control; 3) shared decision-making; 4) identity; and 5) enabling others with similar conditions [13, 19]. To promote empowerment, person-centred care (PCC) is fundamental [20], along with skills in adolescent health and communication [21–23]. PCC means seeing the patient as a capable person with resources, needs and a unique personal narrative, placing this narrative at the centre of care [24]. The transition programme that spanned over a period of 2.5 years (age 16-18.5) was developed into eight key components (i.e. the foundational stones) delivered through five implementation steps (Fig. 1) carried out by a specially trained clinical nurse specialist (i.e. transition coordinator - TC) working at each intervention centre [10, 18].

### Sample and setting

Inclusion criteria were adolescents and parents who participated in the STEPSTONES project and randomized to the intervention group in one of the two intervention centres [10]. We employed a purposive sampling strategy with maximum variation [25] in regard to complexity of the CHD according to standard classification [26], sex and intervention centre. In addition, we endeavoured to sample participants who varied in participation in the implementation steps of the programme (i.e. outpatient visits and adolescent day). Adolescents and parents were approached for participation by text message or letter after they had participated in the programme. Of the 67 adolescents who participated in the intervention group of the RCT, 27 were approached for participation in the present study. Ten adolescents did not answer the text message after several reminders and three declined due to time constraints. The remaining 14 adolescents agreed to participate and fulfilled the study. Regarding the parents, 20 were approached, seven did not answer despite reminders and one declined. The remaining 12 parents accepted and fulfilled the study.

### Data collection

Semi-structured interviews were carried out from October 2019 – October 2020 by the first author (MS) with adolescents approximately two-four months after completion of the programme (i.e. age 18-19). An interview guide was developed based on the components and implementation steps of the transition programme, including follow-up and probing questions (Additional file 2). The guide was piloted during the first interview and was considered adequate. The interviews were carried out face-to-face (*n*=2) or by telephone (*n*=5) or email (*n*=7) in order for the adolescents to choose the communication medium they were most comfortable using [27, 28]. Face-to-face and telephone interviews were audio-recorded and transcribed verbatim and ranged from 53 to 79 minutes (mean= 61). Email-interviews were carried out through two emails, one initial email containing the main questions and one email with follow-up and probing questions.

Parental interviews were carried out from November – December 2020 by two data collection officers (DCO) working as registered nurses at the paediatric cardiology unit in centre 2. The interview guide focused on the parents’ experiences of the transition programme (Additional file 2). As before, the guide was piloted and considered adequate. The 12 interviews were performed over telephone or video-link (*n*=10) and email (*n*=2). Telephone and video-link interviews were audio-recorded and transcribed verbatim (range= 22 – 47 minutes, mean=30). Email-interviews followed the same procedure as for the adolescents. The number of interviews for both adolescent and parental interviews was continuously discussed among the authors and after the last interviews it was deemed redundant to pursue any additional interviews. This because it was considered that the collected data had provided enough depth and breadth to answer the research objective.

### Data analysis

Data analysis was carried out in the computer-assisted qualitative data analysis software NVivo version 12. The first and last author (MS & ELB) are both experienced in qualitative methods and were responsible for the analysis, although this was discussed concurrently with the co-authors to ensure trustworthiness. Data analysis was conducted in two phases. Phase one corresponded with the first aim, whereby a deductive content analysis was performed according to Kyngäs and Kaakinen (2020). Deductive analysis is suitable for exploring an existing theory or framework within a text [29] - in this case, experiences of the components of the transition programme. An unstructured coding matrix based on these components was developed prior to analysis [29]. The analysis started by reading the transcribed texts several times to get a grasp of the whole, after which codes were identified as constellations of words or sentences answering the aim. Codes were then categorized into the existing categories (i.e. eight components of the programme) in the coding matrix. Phase two corresponded with the second aim. Here, we performed an interpretive analysis to explore the mechanisms of impact. Mechanisms were defined as underlying processes or structures that took place between programme delivery and the occurrence of outcomes of interest [30] i.e. how did the components of the transition program lead to an increase in empowerment. Mechanisms were identified through interpreting findings from phase one. To guide in this analysis, we used the logic model of change of the transition program (Additional file 3). Firstly, we explored if empowerment manifested in the results by investigating whether the five dimensions (i.e. knowledge and understanding, personal control, shared decision-making, identity and enabling others [13]) were described in the adolescents and parents experiences of the transition program. Secondly, if the five dimensions of empowerment was identified in these experiences, we sought to understand how the different components of the transition program generated outcomes (i.e. mechanisms) by categorizing these identified mechanisms leading to empowerment into either process or context mechanisms. This analysis generated a conceptual model on the proposed mechanisms of impact of the transition program.

## Results

Table 1 describes the participating sample. The results are presented according to the two aims of the study.

**Table 1:** Demographic characteristics of the study participants in current study in relation to participants in the transition programme (i.e. intervention group)

Characteristics	ADOLESCENTS		PARENTS
	Participants in current study *n*=14 (%)	Participants in transition programme *n*=67 (%)	Participants in current study *n*=12 (%)
Female sex	6 (43)	30 (44.7)	8 (67)
*CHD complexity*^a^			*Child’s CHD complexity*^a^
Mild	4 (29)	11 (16.4)	2 (17)
Moderate	7 (50)	35 (52.2)	6 (50)
Severe	3 (21)	21 (31.3)	4 (33)
*Centre*			
Centre 1	11 (79)	45 (67.2)	9 (75)
Centre 2	3 (21)	22 (32.8)	3 (25)

^a^CHD complexity categorized according to 2018 AHA/ACC Guideline for Management of Adults with Congenital Heart Disease

## Experiences of participating in a person-centred transition programme

**Fig. 2:**
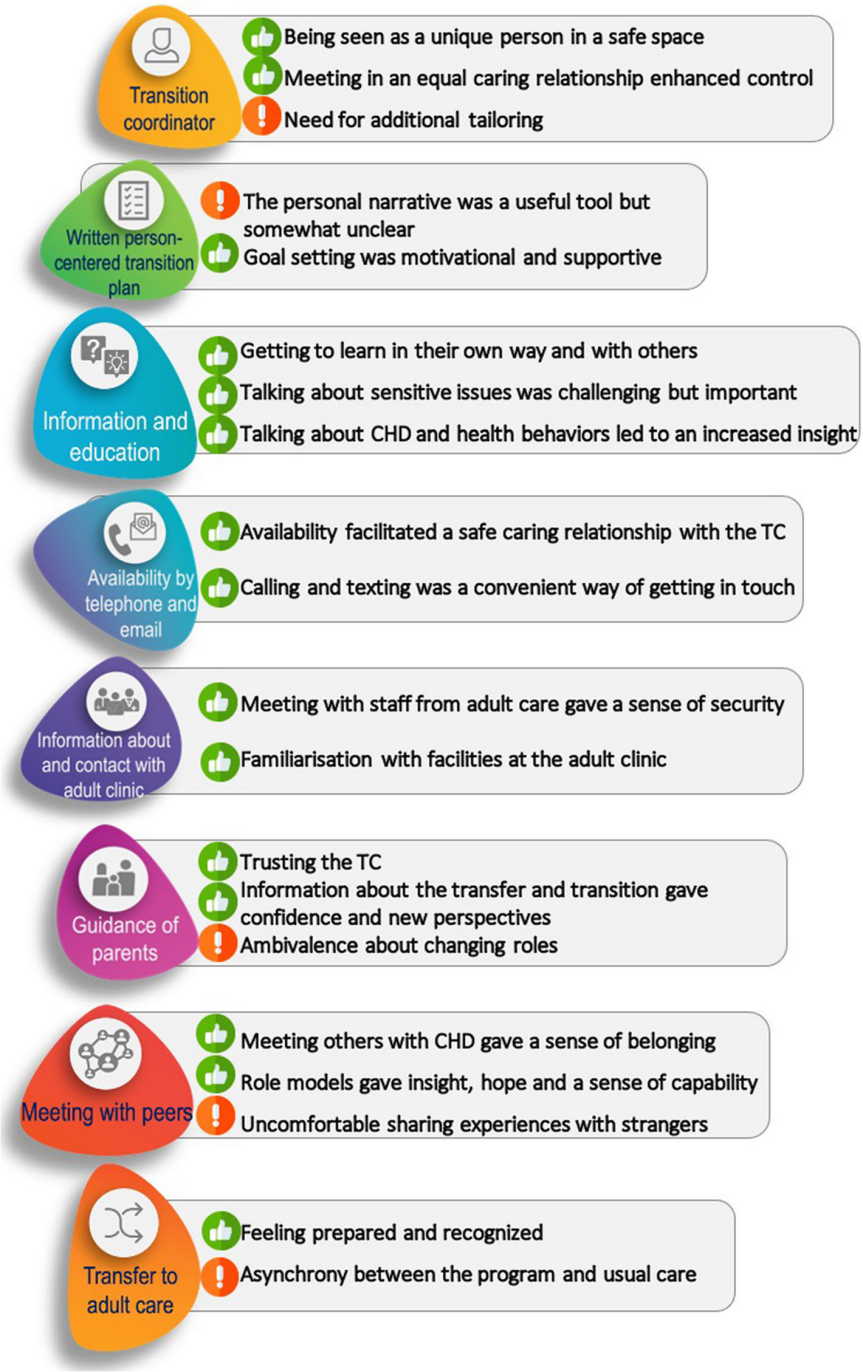
Components and adjacent sub-categories from the deductive content analysis

Figure 2 presents an overview of the components of the transition programme with related subcategories. Results from the parental interviews are only presented in “Guidance of parents” and results from the adolescent interviews are presented in the remaining seven components.

### Transition coordinator

Participation in the transition programme entailed three meetings with the TC at age 16,17 and 18. The adolescents experiences of these meetings and the TC’s role in the programme were characterized by two positive and one constructive aspect.

#### Being seen as a unique person in a safe space

Visiting the TC gave the adolescents the opportunity to talk about their health and CHD, and focus on their personal experiences in a safe space. This resulted in a feeling of being treated as a unique person, as the adolescents had the opportunity to discuss not only issues related to their CHD but also to life in general, such as school, leisure time activities and mental health. Facilitators for opening up about these issues were a warm, friendly and comfortable atmosphere, and the creation of a safe space. Central to this was that participants felt they had sufficient time during their visits and that the TC explicitly stated that the conversations were confidential. Confidentiality and being alone during the TC visits gave the adolescents a sense of trust and security (Table 2, AI 4).

**Table 2:** Illustration of components, subcategories and quotes

Component	Subcategory	Quote
Transition coordinator	*Being seen as a unique person in a safe space*	AI 4: *“When you’re in your teens, you might not want your parents to know everything...I remember she said this is something that will just stay between you and me – these conversations. That the parents wouldn’t be there and she said they didn’t have to know anything about it if you don’t want them to know anything. And that meant I knew I could trust it was like that.”*
	*Meeting in an equal caring relationship enhanced control*	AI 8: *“I was into becoming a blood donor, so we talked a lot about if I could do that…So it felt like I had a lot of control. But it was also very clear that she had things that she needed to bring up too, which was kind of a relief.”*
	*Need for additional tailoring*	AI 1: *“If you would have known that I was not that affected by my heart defect in my everyday life you wouldn’t have had to ask those questions. Partly because it maybe is a waste of your time and resources…To know which people are participating in this study and how much they are affected by their heart defect, I guess. I mean it wasn’t a big thing really, but it felt maybe a bit strange.”*
Written person-centred transition plan	*The personal narrative was a useful tool but somewhat unclear*	AI 8: *“It was good because I still thought it was hard to talk about my heart condition. And it was a real relief I didn’t need to say that when I was there because she had read my narrative before and understood. So that was a real relief. It was nice not to have to mention it yourself.”*
	*Goal setting was motivational and supportive*	AI 3: *“Because it was about me. You feel a bit stupid when you’re going to explain your heart condition and you don’t know anything – you don’t even know what it’s called. So I practised learning the name. And that was more because I felt a bit stupid when people asked...like I didn’t have a clue about my own body or something.”*
Provision of information and education about condition, treatment and health behaviours	*Getting to learn in their own way and with others*	AI 8: *“I liked seeing it in front of me. It was like, very clearly presented. There was a clear list of things that were bad or could involve a risk... so just seeing it in front of you was like...because I’m very visual, so it was really great to see it right there, otherwise it gets very abstract.”*
	*Talking about CHD and health behaviours led to increased insight*	AI 10: *“It feels very important. I mean it’s still a part of me and my growing up and my birth. Yet it hasn’t been a really big part of my life because I’ve never needed surgery since I was born and I’ve only gone for check-ups every four years. So it’s not something you talk about a lot. But it still felt important. It’s very important for me to know.”*
Availability by telephone and email	*Availability facilitated a safe caring relationship with the TC*	AI 10: *“So she was really helpful and said I can call her whenever, if something happens...I got really anxious once, so I called her and it felt much better because she was really supportive... So she really took it very seriously. So, I feel particularly safe knowing that you can turn to someone if you get worried or if something happens.”*
Information about and contact with the adult clinic	*Meeting with staff from adult care gave a sense of security*	AI 8:*” It was nice to see them, that they will be there for me when it’s time. Like even meeting the social worker…That it will not be the first time I will meet the doctor when I go to my first visit.”*
	*Familiarisation with facilities at the adult clinic*	AI 4: *“It was really good that I got to go in and took a look around the building and stuff. I go to the adult clinic by myself now and it feels good because I kind of know where I’m going... to know where I’m going. Otherwise, you can easily feel very lost.”*
Guidance of parents	*Trusting the TC*	*PI 9:” I didn’t expect any support either…I haven’t thought of it like that, that I would receive support in my role as a parent…It feels like I haven’t been that involved…that this was his thing. I haven’t perceived that I should have a role in this.”*
	*Information about the transfer and transition gave confidence and new perspectives*	PI 7: *“Now you are forced to start thinking ‘Do I think that she takes responsibility in this?’ It makes you think…So we as parents have started to discuss this more ‘What do you think?’ ‘Oh, do we think differently?’ So it has affected us a bit”*
	*Ambivalence about changing roles*	*PI 3: “That he still takes responsibility when he feels ill, like last summer, that he actually calls the ambulance and goes to the hospital and stuff. Then he calls and tells us... That he’s grown, that perhaps he knows more about what to do and like he’s learnt to listen to his body himself. And that might have been a combination of both us as parents doing a good job and hopefully, that he got to meet the TC a few times and might have learnt something there.”* *PI 6: “I think both my partner and I have felt that the feedback to us could have been a little better... but I mean, the way these meetings and information evenings have been set up – there’s nothing wrong with that. But I think they could have perhaps given us feedback a little more and more often...to the parents.”*
Meeting with peers	*Meeting others with CHD gave a sense of belonging*	AI 14: *“Another take home for me is these wonderful friends I now have, who can relate to the heart condition so to speak, which other people might not be able to.”*
	*Role models gave insight, hope and a sense of capability*	AI 2: *“They were very calm and had kind of come to terms with their heart condition. So it felt like their way of looking at their heart condition was exactly like mine. That there’s something wrong there but that it won’t prevent me from living my own life. It felt like they knew and that you could identify with it. Definitely.”* AI 8: *“I remember I had an issue with my scars before. I still have some days. And I remember talking to her about it... and she said that you distance yourself to it eventually. You get used to it and learn to live with it. And it was a bit of a relief to hear there were people who had progressed a bit further than me. So, you can get to thinking if it turned out okay for them, it’ll be okay for me too.”*
Transfer to adult care	*Feeling prepared and recognized*	AI 3: *“They were so sweet and prepared and we got to sit down and I got to ask all my questions. And even though I didn’t have that many questions, just the feeling that she took the time for me and allowed me to ask my silly questions was very nice.”*
	*Asynchrony between the programme and usual care*	AI 9: *“I got an appointment at the adult clinic before the study was finished, so I had already met the staff there and therefore I only met with the TC during the last visit. So, my transfer visit didn’t go as planned because I had already met them before I had my last check up in paediatric care. It was a bit weird but I had no problems with it.”*

*AI* adolescent interview, *PI* parent interview

#### Meeting in an equal caring relationship enhanced control

Participants described being in an equal caring relationship with the TC throughout the transition programme. A starting point for this was that the adolescents met with the same healthcare provider on each visit - not different providers, which was the usual care experience. Continuity helped form the basis of the caring relationship and gave the adolescent the courage to ask questions. One facilitator in this respect was that the TC was perceived as informed, humble and able to answer questions and explain complex medical issues in a simple and understandable way, starting from the adolescents’ preunderstanding. As a result, many participants described having control over the conversation and their care within the programme (Table 2, AI 8).

#### Need for additional tailoring

Some participants described feeling inadequately supported in their journey towards independence and wanted more guidance from the TC early on in this process Moreover, for individuals who did not feel afflicted by their CHD in everyday life, some conversations with the TC felt less relevant. From the participants’ point-of-view, this could potentially be resolved by the TC being more informed about the impact of CHD on the individual’s everyday life before their first visit (Table 2, AI 1).

### Written person-centred transition plan

The transition plan, that worked as a documentation within the transition programme, was jointly documented by the TC and adolescent. This plan focused on the adolescents’ goals, needs and arrangements regarding the transfer to adult care and transition to adulthood.

#### The personal narrative was a useful tool but somewhat unclear

Before their first visit, the adolescents wrote a personal narrative describing how they viewed themselves, their health, life and opportunities, and what support they needed from others in relation to their health. Writing this narrative was considered somewhat difficult and unclear in terms of purpose, prompting the adolescents to request more information about how the narrative would be used and what aspects of themselves they should include prior to writing. Nevertheless, the personal narrative was viewed as a useful tool in the conversations with the TC, as it helped the adolescents raise issues in writing that could be difficult to address verbally (Table 2, AI 8).

#### Goal setting was motivational and supportive

To develop increased empowerment, the TC and adolescents worked together to create personal goals for their health in transfer and transition. For many participants, goal setting was considered a natural and expected way of developing themselves in relation to their objectives. Goal setting was seen as a motivator to increase learning about their CHD and other aspects important to their health. The adolescents viewed the TC’s role as important in helping them identify and formulate a goal. However, as setting goals for healthcare was new to most participants, some consideration and discussion was needed in order to establish a relevant and reachable goal. Moreover, even though they recognised the TC as a facilitator, some viewed goal setting as unclear and would have required additional support in order to understand the relevance.

A recurring aspect of working with and reaching the goal was its relevance in relation to the adolescent as a person. Relevance was described as a moderator of how well they worked with the goal and implemented the practices into daily life. For those who saw the goal as relevant, it was commonly formulated as their ability to understand and explain their CHD. Relevant goals could also be related to the adolescent’s general health and wellbeing, for instance physical activity and how to get in touch with healthcare.

To work with these goals, strategies related to the adolescents’ strengths and abilities were implemented. The TC played an important role in enforcing these strengths and abilities and how they could be used to work with and follow up the goal.

In general, all the interviewed adolescents considered that they had reached at least one of the goals formulated in the transition plan. Facilitators for reaching the goals were related to several aspects. Firstly, having a goal that was meaningful and motivating for them as a young person living with a CC was important (Table 2, AI 3). Having a goal that felt achievable also gave self-confidence when it was achieved. The need to show others in their surroundings, such as family and friends was another facilitator. Barriers to reaching the goal were related to fear or discomfort, that the goal felt unreachable or not important enough, and that the adolescent could have needed more support in working with the goal. Moreover, some adolescents felt that their parents were a resource and would have wanted to include them more in relation to goal setting.

### Provision of information and education about condition, treatment and health behaviours

Throughout the different implementation steps of the transition programme, information and education about the adolescents’ CHD, treatment and health behaviours were provided.

#### Getting to learn in their own way and with others

A central aspect of the learning process was that learning was active and tailored to the adolescent’s personal preferences and learning style. Learning was achieved through the use of visual aids, such as anatomic figures of the heart, pictures and websites that provided films and animations of their CHD. This resulted in the feeling of being able to learn difficult and complex topics in a personalized and understandable way (Table 2, AI 8).

Moreover, being given the opportunity to ask the same questions on repeated occasions (i.e. visits with TC and information days) made it easier for some participants to gain deeper understanding. In addition, the social aspect of the learning process was considered important. Learning through discussions, hearing your peers’ questions and conversations between participants during the information day enhanced learning.

#### Talking about sensitive issues was challenging but important

Talking about sensitive issues evoked a range of feelings in the adolescents. Mostly, talking about sex, drugs and alcohol habits was described as a relief because many of the participants had limited knowledge about how these habits affected their CHD. Many also felt that this information was timely and relevant, as it was considered a normal part of adolescent life. Nevertheless, some participants felt uncomfortable discussing these topics as they were considered taboo or they were not sexually active. However, the TC’s open-minded approach when raising these subjects was considered a facilitating aspect, as it made the adolescents feel safe in talking about these issues. Female participants also felt it was easier to discuss these issues with someone of the same gender.

#### Talking about CHD and health behaviours led to increased insight

Participants described several learning outcomes related to their CHD and health. For example, the information and learning process in the transition programme were considered to be on a much deeper and detailed level than that provided in usual care. Potential reasons for this were that the adolescents felt their own views were at the centre of care, that the TC took time to actively listen, and that the adolescents’ prior knowledge was the starting point of the educational process. Talking about their CHD and health behaviours led to several insights. Firstly, the adolescents recognised that they had to manage their health and care without their parents in adult life. Secondly, they understood why they had to remain in follow-up for their CHD after transfer to adult care. Thirdly, as the adolescents were expected to be independent in adult life, they had to develop adequate communication skills to explain their CHD to others. Fourthly, they learned about important lifestyle issues in adult life, such as vocational and educational aspects when living with a CC. Finally, the conversation led to the adolescent feeling that they could place their care and treatment in relation to themselves and importance in their lives (Table 2, AI 10). However, the information provided in the transition programme did not cover all aspects, which some participants identified a shortcoming. Two aspects were emphasized: the need for more tailored information to my specific situation, and that the information delivered did not contribute with any new knowledge.

### Availability by telephone and email

The TC extended their availability by providing a personal telephone number and the opportunity for the participants to use text messaging and email.

#### Availability facilitated a safe caring relationship with the TC

The opportunity to call, text or email the TC facilitated a sense of safety and comfort in the caring relationship. Availability made it easier to have a more direct and personal relationship with the TC through a communication medium that they were comfortable using. Some made use of this opportunity to get in touch with the TC when they needed support (Table 2, AI 10). Although some adolescents did not express a need to have additional contact with the TC in-between visits, knowing that the TC was available enforced their sense of security and comfort.

#### Calling and texting was a convenient way of getting in touch

Being able to choose your preferred communication medium when contacting the TC was both practical and gave a sense of control. The text messaging was considered the most convenient way of getting in touch for questions in general.

### Information about and contact with the adult clinic

This component was delivered through the three visits with the TC and the information day for adolescents and parents.

#### Meeting with staff from adult care gave a sense of security

Being able to meet nurses and physicians from the adult outpatient clinic during the information day and transfer meeting gave a sense of security. The transfer from paediatric to adult care was depicted as a life-changing event, clouded by insecurity. Getting to meet the adult staff and seeing their competence and engagement was comforting. Meeting staff made it easier for the adolescents to cope with emotions related to the fact that their parents were no longer a natural part of their care. Furthermore, new insights were also gained from meeting staff from the adult clinic, including information about what other professions (e.g. social workers and physiotherapists) could provide for the adolescents. This type of information was perceived to be lacking in paediatric care (Table 2, AI 8).

#### Familiarisation with facilities at the adult clinic

A complementary aspect to meeting the adult staff was familiarisation with the practical aspects of transfer to adult care, which included visiting the adult clinic prior to the transfer and seeing the facilities. This promoted a feeling of safety in knowing where the clinic was and alleviated any worries prior to the first follow-up visit after transfer (Table 2, AI 4).

### Guidance of parents

As highlighted by the interviews, parents were involved in the programme to a different extent than their children. Most commonly parents were invited to take part in the first visit with the TC and during the information day.

#### Trusting the TC

All the participating parents considered their children to be in safe and trustworthy hands with the TC as a healthcare professional, but many did not see the TC as a support for themselves as parents and did not request additional support – it was enough to know the TC was available if needed. In general, parents saw their role in the transition programme as one of supporting their child and that it was appropriate for parents to have limited participation (Table 2, PI 9).

#### Information about the transfer and transition gave confidence and new perspectives

Parents felt the information about transfer, transition and their children’s care was sufficient for their own needs as parents and tailored to their personal preferences. Having the opportunity to ask questions in a group setting during the information day and meet others in the same situation was also brought up as important. A key feature of the information day was the opportunity to meet staff from adult care, which gave parents confidence in their children’s future care, as they could see that the adult care system was adequately organized to cater to their child’s needs. One effect of receiving this information was that the parents made transition a part of their family discussion agenda (Table 2, PI 7).

#### Ambivalence about changing roles

Although parents generally viewed the programme in a positive light they struggled with mixed emotions regarding transfer to adult care, transition to adulthood and the impact of the transition programme on themselves. This resulted in ambivalence about changing roles, especially in their adolescent’s child’s life. For many, the programme helped them in shift from a feeling of insecurity to security, some even stating that it helped them gradually hand over health and care responsibility to their adolescent. Knowing that their children were more prepared after participating in the programme was a facilitator in this process. Parents described it as a process of learning how to both step back and let go which, for some resulted in their becoming a support system, rather than a caregiver (Table 2, PI 3). Nevertheless, many felt a lack of participation in their children’s care, which was considered a shortcoming of the programme. In general, parents requested more information about their children’s care throughout the programme and some participants felt they were losing control and being left out. Moreover, many felt they lacked closure or a final meeting with paediatric care, which would have involved them in the transition plan and given them the opportunity to ask medical questions, for example, regarding the need for future interventions and prognosis (Table 2, PI 6).

### Meeting with peers

Adolescents and parents on the transition programme were able to meet each other during the information day to share thoughts and concerns regarding the transfer and transition, adolescent life with CHD and parenting a child in transition to adulthood. Two young adults with CHD also participated during this meeting, acting as facilitators and sharing their experiences.

#### Meeting others with CHD gave a sense of belonging

For many, the information day was the first time they had met another person with CHD. The informal nature of the meeting made the adolescents more comfortable about taking the opportunity to chat and share experiences. Hearing their peers’ life stories and thoughts sparked recognition and made the participants feel less lonely. Although not all stories were universal or relatable, the accounts were still seen as a strength, as they gave the adolescents new perspectives on their own situation. For some, the outcome of meeting their peers was the formation of new friendships and online communities. These peer communities acted as a help in symptom management, alongside with contact with the CHD patient organization (Table 2, AI 14).

#### Role models gave insight, hope and a sense of capability

Having two young adults with CHD involved during the information day prompted a variety of reactions. Most participants saw them as role models and as the most impactful aspect of this component because they were perceived as a reliable source of information about the experience of transfer and transition and the management of CHD in young adulthood (Table 2, AI 2). Another aspect of the young adults’ contribution was to give the adolescents hope and a sense of capability – that even though CHD was a limiting factor, they could still live fulfilling lives. The young adults also shared experiences that were considered tacit knowledge and that the healthcare professionals might have been unaware of (Table 2, AI 8). Nevertheless, not all participants viewed the young adults as role models, one reason being that they felt they could not relate to the young adults’ experiences. This was mainly because the adolescents thought their CHD was not as serious as the young adults’.

#### Uncomfortable sharing experiences with strangers

During the information day, the group dynamic affected some participants’ willingness to share experiences. This included the perception that the other participants were shy, passive and uninterested, that there were too few participants to have a fruitful discussion, and that the other participants felt more hampered by their CHD. In general, these aspects affected the willingness to share and discuss experiences.

Moreover, not all the interviewed adolescents chose to participate in the information day and there were three main reasons for this. Firstly, they felt the information they received from the TC about the information day indicated they were obliged to share experiences about themselves with the other participants. For some, sharing personal experiences felt uncomfortable or strange, as they considered themselves unaffected by their CHD. Secondly, meeting strangers was seen as awkward. And thirdly, the participants stated lack of time or other commitments.

### Transfer to adult care

The final component of the transition programme was the joint transfer meeting between the adolescent, nurse from adult care and TC.

#### Feeling prepared and recognized

Meeting the nurse from adult care together with the TC made the adolescent feel prepared for the transfer. A positive aspect of the visit was that the nurse was informed about who they were, which led to feeling recognized. In addition, information about the transfer and structure of adult care (including follow-up visits) were discussed, which gave confidence about how the future interactions with adult staff would be (Table 2, AI 3).

#### Asynchrony between the programme and usual care

The participants experienced limitations to this component, mainly related to the administration and cooperation between the paediatric and adult clinic. Participants felt it was difficult to find a suitable time to have the transfer meeting and when the meeting was held, some experienced it as unplanned and that the adult care nurse was not prepared. Moreover, during the transfer meeting there were still questions left unanswered that were considered important, such as information about the physician responsible for the follow-up visits (Table 2, AI 9).

## Mechanism of impact of the transition program

**Fig. 3:**
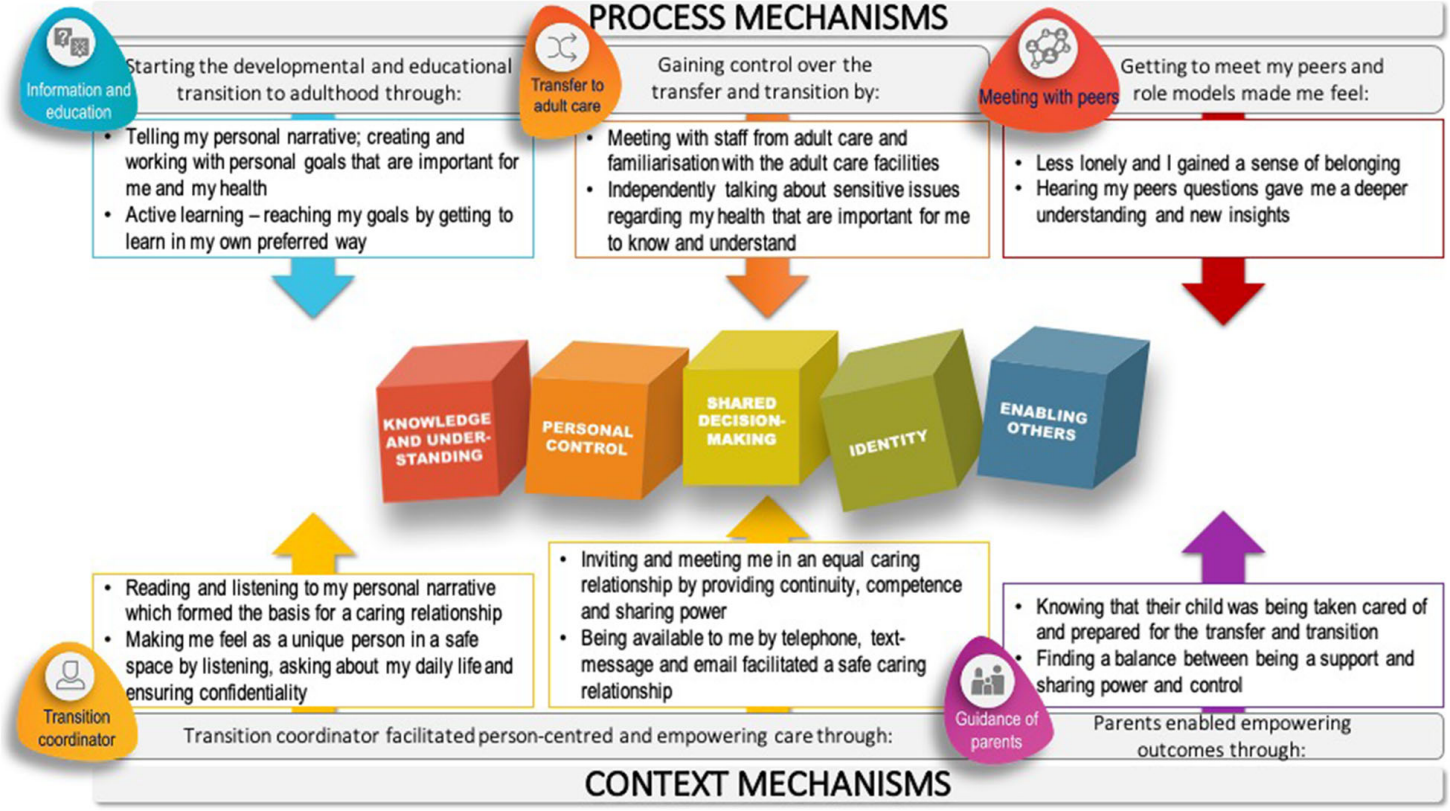
Conceptual model for proposed mechanism of impact of the STEPSTONES transition programme

The analysis generated a conceptual model on the mechanisms of impact of the STEPSTONES transition programme, which program activities (i.e. components) that were responsible for these and the generated outcomes. The mechanisms were categorised to either the process of delivering the program or to the context (Fig. 3). In the dimension of knowledge and understanding, interpretation of results from phase one led to the understanding that the programme activities helped the adolescents become experts on their CHD, and understand how this condition affected them and how to communicate this to others. Example of mechanisms responsible for this outcome were the opportunity to learn about their CHD in a safe space through PCC. Personal control over the transfer to adult care and transition to adulthood led to the ability to manage their care. The mechanism responsible for this was meeting staff from adult care and thus becoming familiar with the physical and relational aspects of the transfer. Shared decision-making was facilitated through several mechanisms related to the power exchange between the adolescent and TC. For instance, the TC’s extended availability made the adolescents feel they could meet in an equal caring relationship. Finally, identity and the ability to enable others were achieved by getting to meet others in the same situation. Meeting others made the adolescents feel less lonely, that they were part of a community, and generated increased hope about adult life.

## Discussion

Process evaluations aim to explore which components of the intervention are more important in achieving the outcome [9]. Our study has identified several components considered pivotal in this achievement. However, the mechanisms are often the result of synergistic effects between components in a particular context, and this needs to be considered when understanding the findings [14, 30]. The most important contextual mechanisms were those created by the TC which interacted with most components in the programme. Our findings showed that continuity, confidentiality and being able to talk about sensitive issues in a safe space were pivotal for the adolescents. These needs have been expressed in preparatory studies [31–33] and are considered an essential part of adolescent health [22, 23, 34]. Moreover, as empowerment can be seen as both a process and an outcome [13] the creation of an equal caring relationship that builds on PCC is an example of an empowering process where shared-decision making was one of the outcomes. Furthermore, health-related learning was evident in the results. Goal setting was most often related to learning about one’s CHD and how to communicate this to others. This finding might be especially common to adolescents with CHD, where disease-related knowledge is low [31, 35, 36]. However, the educational process and the provision of tailored learning strategies were a central aspect of this achievement. Indeed, *active learning* was one behaviour-change technique selected in the intervention development phase [18], indicating that this component was delivered successfully. Another important finding was the meaning of peer support. This component was experienced by many as most impactful. Transition interventions in other patient populations have shown similar results [37], highlighting the importance of meeting others with similar conditions. However, our results showed that there were some limitations in the delivery of this component. For instance, several participants did not attend the information day due to feeling uncomfortable about interacting with other adolescents’ with CHD. Previous studies have shown that adolescents lack of engagement in their illness affects study participation negatively [15]. In addition, group dynamic and not being able to identify with the role models during the information day were seen as shortcomings. Indeed, for role models to be effective the participant has to be able to identify with them [38, 39]. As the CHD population is diverse in regard to disease complexity and longevity throughout the life course [40], role models used in future transition interventions should consider this diversity.

Our findings showed that although most components were experienced positively and achieved increased empowerment, some were delivered insufficiently or did not receive the desired response. Parents did not consider themselves involved in the programme, which for some resulted in ambivalence about changing roles. As parents are an important resource in the adolescents’ transition to adulthood [41, 42] this can be considered as a shortcoming in the implementation of the programme. However, not all parents expressed the need for additional guidance. Tailoring to the family’s needs is therefore important for future studies. Another component that was poorly implemented according to the findings was the transfer meeting with adult care. A reason for the experienced asynchrony between the intervention and usual care might be that follow-up visits are defined according to CHD complexity, ranging from one to every three to five years [26]. Thus, the follow-up visit in usual care did not always synchronize with the transfer meeting in the intervention. Our findings therefore emphasize the need for a more person-centred approach towards transfer and transition, whereby follow-up visits are based on the patients’ health needs in addition to medical parameters.

Some methodological limitations must be addressed. Thirteen out of 27 adolescents and 8 out of 20 parents declined participation or were unreachable. The transferability of findings in relation to possible selection bias must therefore be considered, as the sample may have had more positive experiences of the intervention. The sample was nevertheless representative of the whole group of participants in the transition programme and had a variation in participation in the implementation steps of the program (Table 1). The use of various forms of interviews gave participants the opportunity to select their preferred medium of communication [27, 28] meaning a wide range of perspectives on the intervention were gathered. Recall bias may be considered another limitation, as the intervention period was 2.5 years and the interviews were performed after participation. However, the timing of interviews was appropriate, as performing them earlier may have affected the effectiveness evaluation due to this study being based on experiences and mechanisms leading to outcomes. We used method triangulation to deal with recall bias in our process evaluation [9, 43]. By using participatory observations, interviews and quantitative assessments of the components and implementation steps we aim to capture different aspects of the intervention in future studies [11].

In light of these limitations, our study has several strengths. Firstly, the use of intervention mapping (a proven intervention development framework) [44] facilitated the successful delivery of an intervention that reached the performance objectives stated in the logic model [18]. Moreover, the outcomes presented in this study respond to findings from the preparatory studies, which ensures credibility and transferability of our results [45]. Secondly, the findings of this study were analysed and reported before knowing the outcome of the effectiveness evaluation, thus avoiding biased interpretation of future findings [9]. As a result, this study generates a hypothesis on how the intervention worked and can shed light on mechanisms responsible for working towards the outcome, as well as components that were implemented poorly and can hamper outcomes. Thirdly, as parents have a central role in the transition [46, 47], including them in this study has identified moderating factors on the mechanisms of the transition programme and highlighted components that were insufficiently delivered. Moreover, using different interviewers for adolescent and parent interviews (i.e., investigator triangulation) allowed us to handle bias associated with using one principal interviewer [43], thus increasing dependability and credibility of findings [45]. Finally, by sampling participants with varied participation in the components and implementation steps of the programme, we gained perspectives on how the programme and its components were perceived. These findings are especially important for the implementation of transition programmes in other settings and populations.

## Conclusion

This is the first study exploring the mechanisms of impact of a transition programme for adolescents with CC. Participants experienced increased empowerment in several dimensions of this construct and the change mechanisms have been illuminated. Consequently, a transition programme consisting of a transition coordinator trained in PCC and adolescent health, an educational process starting from the adolescents’ prerequisites and preferences and peer support are central aspects for empowering care in transition to adulthood. We should note, however, that support for parents was not delivered to a great extent. Although not all parents requested support, there were some with unmet needs that could have been addressed. For future implementation of transition programmes, these findings can inform how evidence-based behaviour-change techniques work in practice, as well as the challenges associated with delivering a complex intervention in combination with usual care.

## Supplementary Information

**Additional file 1.** Consolidated criteria for reporting qualitative studies (COREQ): 32-item checklist.

**Additional file 2.** Interview guide, adolescent interviews.

**Additional file 3.** Logic model of change.

## Data Availability

All data and materials are available from the corresponding author upon request.

## Abbreviations

CC: Chronic conditions
CHD: Congenital heart disease
COREQ: Consolidated criteria for reporting qualitative studies
DCO: Data collection officer
I: Interview
PCC: Person-centred care
RCT: Randomized controlled trial
STEPSTONES: Swedish Transition Effects Project Supporting Teenagers with chrONic mEdical conditionS
TC: Transition coordinator
