# Supplementary material for: Mechanisms of impact and experiences of a person-centred transition programme for adolescents with CHD: the Stepstones project
Source: BMC Health Serv Res. 2021 Jun 10;21:573. doi: 10.1186/s12913-021-06567-1 (PMC8194131; doi:10.1186/s12913-021-06567-1)
Supplement: Supplementary file 1 — Additional file 1. Consolidated criteria for reporting qualitative studies (COREQ): 32-item checklist. [file 12913_2021_6567_MOESM1_ESM.docx]

**ADDITIONAL FILE 1**

**CONSOLIDATED CRITERIA FOR REPORTING QUALITATIVE STUDIES (COREQ): 32-ITEM CHECKLIST**

# **MECHANISMS OF IMPACT AND EXPERIENCES OF A PERSON-CENTRED TRANSITION PROGRAMME FOR ADOLESCENTS WITH CHD: THE STEPSTONES PROJECT**

*Markus Saarijärvi MSc, RN^1, 2^, Lars Wallin PhD, RN^1, 3^, Philip Moons PhD, RN^1-2, 4^, Hanna Gyllensten PhD, RPH^1, 5^, Ewa-Lena Bratt PhD, RN^1, 6^*

**Authors’ information**

^1^ Institute of Health and Care Sciences, University of Gothenburg, Gothenburg, Sweden, ^2^ KU Leuven, Department of Public Health and Primary Care, Leuven, Belgium, ^3^ School of Education, Health and Social Studies, Dalarna University, Falun, Sweden, ^4^ Department of Paediatrics and Child Health, University of Cape Town, Cape Town, South Africa, ^5^ University of Gothenburg Centre for Person-Centred Care (GPCC), Sahlgrenska Academy, University of Gothenburg, Gothenburg, Sweden, ^6^ Department of Pediatric Cardiology, The Queen Silvia Children’s Hospital, Gothenburg, Sweden.

**Corresponding author**

Markus Saarijärvi, Institute of Health and Care Sciences, University of Gothenburg, Sweden. E-mail: [markus.saarijarvi@gu.se](mailto:markus.saarijarvi@gu.se). Tel: +46 739 29 20 88.

Developed from:

Tong A, Sainsbury P, Craig J. Consolidated criteria for reporting qualitative research (COREQ): a 32-item checklist for interviews and focus groups. *International Journal for Quality in Health Care*. 2007. Volume 19, Number 6: pp. 349 – 357

| **No. Item** | **Guide questions/description** | **Reported on Page #** |
| --- | --- | --- |
| **Domain 1: Research team and reﬂexivity** |  |  |
| *Personal Characteristics* |  |  |
| 1. Inter viewer/facilitator | Which author/s conducted the interview or focus group? | Methods, p5 |
| 2. Credentials | What were the researcher’s credentials? E.g. PhD, MD | Methods, p5 and authors information |
| 3. Occupation | What was their occupation at the time of the study? | Methods, p5-6 and authors information |
| 4. Gender | Was the researcher male or female? | Authors information |
| 5. Experience and training | What experience or training did the researcher have? | Methods, p5-6 |
| *Relationship with participants* |  |  |
| 6. Relationship established | Was a relationship established prior to study commencement? | N/A |
| 7. Participant knowledge of the interviewer | What did the participants know about the researcher? e.g. personal goals, reasons for doing the research | Ethical considerations |
| 8. Interviewer characteristics | What characteristics were reported about the inter viewer/facilitator? e.g. Bias, assumptions, reasons and interests in the research topic | N/A |
| **Domain 2: study design** |  |  |
| *Theoretical framework* |  |  |
| 9. Methodological orientation and Theory | What methodological orientation was stated to underpin the study? e.g. grounded theory, discourse analysis, ethnography, phenomenology, content analysis | Methods, p4 and 6 |
| *Participant selection* |  |  |
| 10. Sampling | How were participants selected? e.g. purposive, convenience, consecutive, snowball | Methods, p5 |
| 11. Method of approach | How were participants approached? e.g. face-to-face, telephone, mail, email | Methods, p5 |
| 12. Sample size | How many participants were in the study? | Methods, p5-6 and  Results, table 1 |
| 13. Non-participation | How many people refused to participate or dropped out? Reasons? | Methods, p5 |
| *Setting* |  |  |
| 14. Setting of data collection | Where was the data collected? e.g. home, clinic, workplace | Methods, p5-6 |
| 15. Presence of non-participants | Was anyone else present besides the participants and researchers? | N/A |
| 16. Description of sample | What are the important characteristics of the sample? e.g. demographic data, date | Methods, p5-6 and Results table 1 |
| *Data collection* |  |  |
| 17. Interview guide | Were questions, prompts, guides provided by the authors? Was it pilot tested? | Methods, p5-6 and Additional file 2 |
| 18. Repeat interviews | Were repeat interviews carried out? If yes, how many? | Methods, p5-6 |
| 19. Audio/visual recording | Did the research use audio or visual recording to collect the data? | Methods, p5-6 |
| 20. Field notes | Were ﬁeld notes made during and/or after the inter view or focus group? | N/A since this is not relevant for the methodology |
| 21. Duration | What was the duration of the inter views or focus group? | Methods, p5-6 |
| 22. Data saturation | Was data saturation discussed? | Methods, p5-6 |
| 23. Transcripts returned | Were transcripts returned to participants for comment and/or correction? | No |
| **Domain 3: analysis and ﬁndings** |  |  |
| *Data analysis* |  |  |
| 24. Number of data coders | How many data coders coded the data? | Methods, p6-7 |
| 25. Description of the coding tree | Did authors provide a description of the coding tree? | Results, Table 2 |
| 26. Derivation of themes | Were themes identiﬁed in advance or derived from the data? | Methods, p6-7 |
| 27. Software | What software, if applicable, was used to manage the data? | NVivo (p6) |
| 28. Participant checking | Did participants provide feedback on the ﬁndings? | No |
| *Reporting* |  |  |
| 29. Quotations presented | Were participant quotations presented to illustrate the themes/ﬁndings? Was each quotation identiﬁed? e.g. participant number | Results, Table 2 |
| 30. Data and ﬁndings consistent | Was there consistency between the data presented and the ﬁndings? | Results and discussion |
| 31. Clarity of major themes | Were major themes clearly presented in the ﬁndings? | Results, figure 2 and 3 |
| 32. Clarity of minor themes | Is there a description of diverse cases or discussion of minor themes? | Discussion |
