# Supplementary material for: Mechanisms of impact and experiences of a person-centred transition programme for adolescents with CHD: the Stepstones project
Source: BMC Health Serv Res. 2021 Jun 10;21:573. doi: 10.1186/s12913-021-06567-1 (PMC8194131; doi:10.1186/s12913-021-06567-1)
Supplement: Supplementary file 2 — Additional file 2. Interview guide, adolescent interviews. [file 12913_2021_6567_MOESM2_ESM.docx]

**ADDITIONAL FILE 2**

# **MECHANISMS OF IMPACT AND EXPERIENCES OF A PERSON-CENTRED TRANSITION PROGRAMME FOR ADOLESCENTS WITH CHD: THE STEPSTONES PROJECT**

*Markus Saarijärvi MSc, RN^1, 2^, Lars Wallin PhD, RN^1, 3^, Philip Moons PhD, RN^1-2, 4^, Hanna Gyllensten PhD, RPH^1, 5^, Ewa-Lena Bratt PhD, RN^1, 6^*

**Authors’ information**

^1^ Institute of Health and Care Sciences, University of Gothenburg, Gothenburg, Sweden, ^2^ KU Leuven, Department of Public Health and Primary Care, Leuven, Belgium, ^3^ School of Education, Health and Social Studies, Dalarna University, Falun, Sweden, ^4^ Department of Paediatrics and Child Health, University of Cape Town, Cape Town, South Africa, ^5^ University of Gothenburg Centre for Person-Centred Care (GPCC), Sahlgrenska Academy, University of Gothenburg, Gothenburg, Sweden, ^6^ Department of Pediatric Cardiology, The Queen Silvia Children’s Hospital, Gothenburg, Sweden.

**Corresponding author**

Markus Saarijärvi, Institute of Health and Care Sciences, University of Gothenburg, Sweden. E-mail: [markus.saarijarvi@gu.se](mailto:markus.saarijarvi@gu.se). Tel: +46 739 29 20 88.

**INTERVIEW GUIDE, ADOLESCENT INTERVIEWS**

1. I’d like to start by asking you to summarise – in your own words – the content of the transition programme.
2. (Like you told me before) So, in these past two and a half years, you’ve met with a transition co-ordinator (co-ordinator’s name) three times. Could you tell me what your experience was of the first visit?
3. What was your experience of the second visit? (possible follow-up questions)
4. What was your experience of the third visit? (possible follow-up questions)
5. How did you think the TC addressed your questions and concerns?
6. Did you feel you had any influence over the discussion?
7. Did you think it was difficult to talk about certain things?
8. Did you feel comfortable talking about things that can be perceived as sensitive? (for example, sex, drugs, alcohol)
9. Can you tell me who was present at these meetings with the TC?
10. Was your visit with the TC different to visits you’ve had with doctors and other nurses at the paediatric heart clinic?
11. Before your first visit with the TC you had to write a personal letter about yourself. What was your experience of that?
12. During the visit with the TC were you allowed to formulate your own goals? How did that feel?
13. How did it feel to be allowed to write these goals down yourself?
14. Can you give an example of a goal you formulated?
15. In what way did the TC follow up the goal you formulated?
16. Do you feel you achieved the goal you formulated?
17. Did you discuss what you spoke about during your meetings with the TC with your doctor or other nurses at the clinic?
18. Do you think the visits in ‘usual healthcare’ – meaning with your usual doctor – that these visits have changed since you started participating in this study?
19. Part of the programme is to receive information and education about your heart condition, treatment and various health habits. Can you describe how this information and education was for you?
20. Were you allowed to choose how you would learn?
21. Did you feel the TC sought out information that was relevant and important to you personally?
22. In what way did the TC teach or inform you?
23. Did you feel the TC was available to answer your questions in between visits?
24. What kind of contact did you have (text message, telephone or email)?
25. What kind of contact did you prefer?
26. What did you talk about on the telephone, by text message or by email?
27. Was it easier to talk about certain things by text message? If so, what things?
28. Did you participate in the information evening?
29. Could you summarise the content of the information evening and tell me what you did?
30. What was your experience of the information evening?
31. How did it feel to sit in a group and talk to other young people?
32. What did you think about the three films that were shown?
33. As you told me before, there were a number of different people there, for example, adult healthcare staff, a wellbeing officer, physiotherapist and two young adults with congenital heart defects. What was your experience of their participation?
34. In regard to the people who were there, which ones do you think were the most important or most meaningful to you?
35. Has your participation affected your relationship with your parents, and if so, how?
36. Do you think participating in the study has prompted your parents to start sharing their experiences with you? Have you started talking more about your heart condition?
37. Do you feel your parents are more secure about handing over responsibility for your health to you, now that you’ve participated in the study?
38. Can you tell me what you thought about your last visit with the nurse from the adult care clinic?
39. You were asked to write a new personal letter to the adult clinic to take with you. Did you do it? How did you feel about that?
40. How does it feel, now that you’ve participated in this study?
41. If you could decide, what would you change or improve about the programme?
42. What is the most important thing you’ve taken away with you from participating in this study?
43. Would you recommend others in a similar situation to participate?
44. Finally, I’d like to ask you. The purpose of the programme is, of course, to make you feel stronger and more secure abut transfer and transition. How do you feel about that? Do you feel stronger and more secure?
45. I have no further questions. Thank you for sharing your experiences with me. Is there anything more you’d like to add or any questions you’d like to ask me before we finish the interview?

**INTERVIEW GUIDE, PARENT INTERVIEWS**

1. I thought we’d start from the beginning and talk a little about when you were asked to participate in the transition programme. Can you tell me how you felt when you were asked to participate? Can you tell me a little about why you chose to accept/participate in the programme?
2. What did you think about the information you received before joining the programme?
3. Would you be able to summarise – in your own words – the content of the transition programme?
4. Based on what you just told me about the programme... can you tell me how you experienced your child’s participation in the programme?
5. What role did you think the transition coordinator played?
6. Can you describe what your role has been in regard to participating in the programme? How active do you feel you have been?
7. What do you think your child has taken home from this programme that they wouldn’t have done, if they only went on their regular visits to the heart clinic (doctor’s appointments etc)?
8. Can you give examples of how your child has started to take more responsibility for their health and care since they joined the programme?
9. Do you feel you the programme has supported you in the change of your parental role?
10. How do you feel now, after participating in this programme?
11. How could the programme be improved, if it is offered to all adolescents with congenital heart defects in the future?
12. What is your take home from participating in the transition programme as a whole?
13. What do you think is the most important thing your child has taken home with them from participating in this programme?
14. I have no further questions. Thank you for sharing your experiences with me. Is there anything more you’d like to add or any questions you’d like to ask me before we finish the interview?
