# Supplementary figures and images for: Mechanisms of impact and experiences of a person-centred transition programme for adolescents with CHD: the Stepstones project
Source: BMC Health Serv Res. 2021 Jun 10;21:573. doi: 10.1186/s12913-021-06567-1 (PMC8194131; doi:10.1186/s12913-021-06567-1)

**
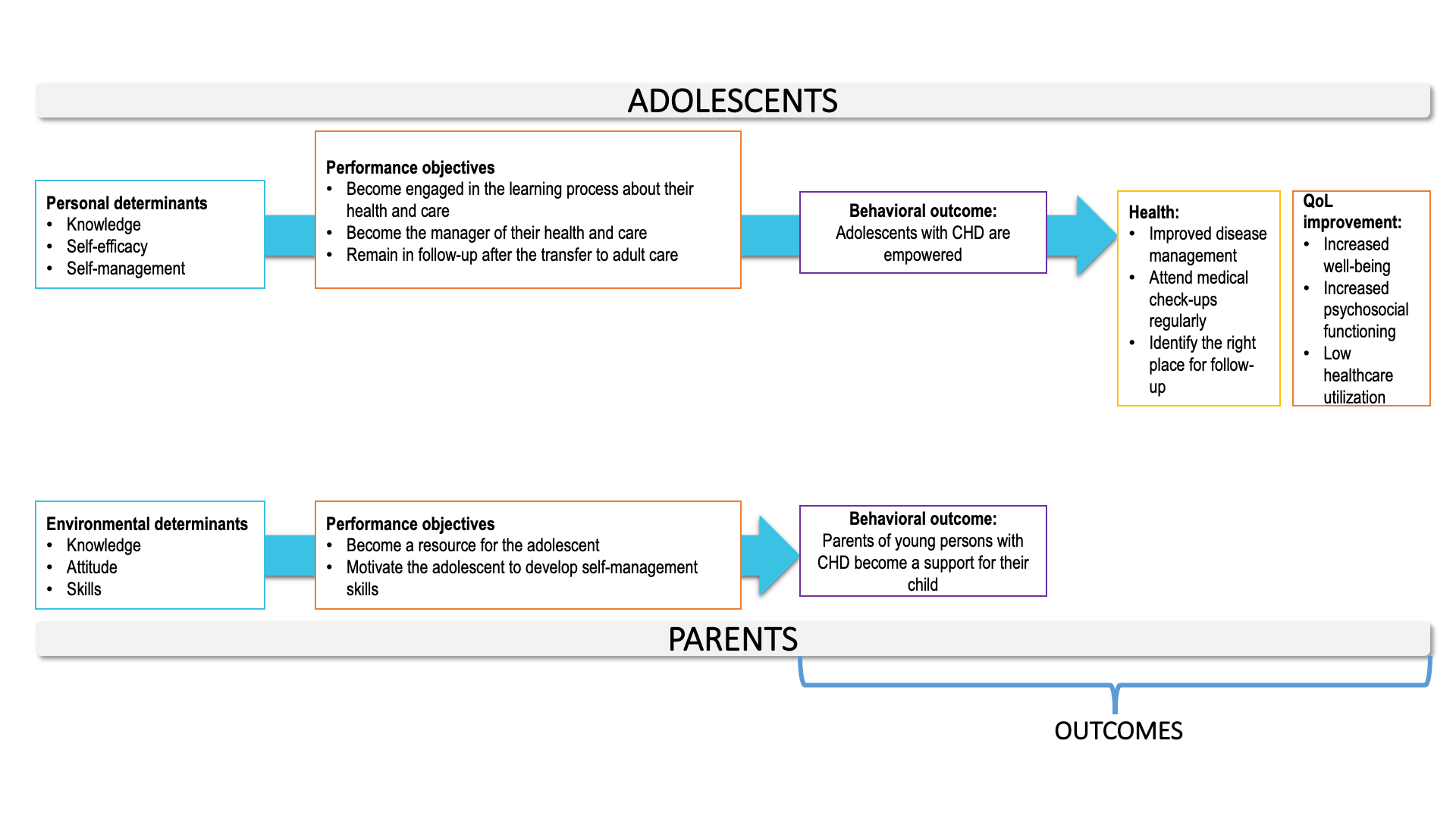
ADDITIONAL FILE 3 – LOGIC MODEL OF CHANGE**

Supplement: Supplementary file 3 — Additional file 3. Logic model of change. [file 12913_2021_6567_MOESM3_ESM.docx]
